# (Acetato-κ*O*)(2,5,5,7,9,12,12,14-octa­methyl-1,4,8,11-tetra­aza­cyclo­tetra­decane-κ^4^ *N*,*N*′,*N*′′,*N*′′′)zinc perchlorate

**DOI:** 10.1107/S1600536811045582

**Published:** 2011-11-05

**Authors:** Tapashi G. Roy, Saroj K. S. Hazari, Kanak K. Barua, Edward R. T. Tiekink

**Affiliations:** a Department of Chemistry, University of Chittagong, Chittagong 4331, Bangladesh; b Department of Chemistry, University of Malaya, 50603 Kuala Lumpur, Malaysia

## Abstract

The Zn^II^ atom in the cation of the title salt, [Zn(C_2_H_3_O_2_)(C_18_H_40_N_4_)]ClO_4_, is five-coordinated by the four N atoms of the macrocycle and the O atom of the monodentate acetate ligand. The N_4_O donor set is based on a trigonal bipyramid with two N atoms occupying axial positions [N—Zn—N = 170.89 (16)°]. The perchlorate anions are associated with the cations *via* N—H⋯O hydrogen bonds; intra­molecular N—H⋯O(acetate) inter­actions are also observed. The neutral aggregates are connected into an helical chain along the *b* axis *via* N—H⋯O(acetate) hydrogen bonds. The perchlorate anion was found to be disordered about a pseudo-threefold axis: the major component of the disorder had a site occupancy factor of 0.692 (11).

## Related literature

For background to the synthesis, characterization, kinetic studies and biological activity of 14-membered methyl-substituted tetra­aza­macrocyclic ligands, their *N*-substituted derivatives and their metal complexes, see: Bembi *et al.* (1990 ▶); Roy *et al.* (2007 ▶, 2011 ▶); Hazari *et al.* (2008 ▶). For additional geometric analysis, see: Addison *et al.* (1984 ▶).
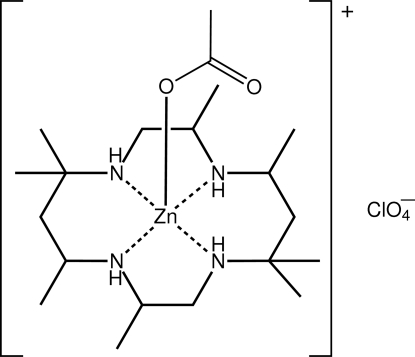


## Experimental

### 

#### Crystal data

[Zn(C_2_H_3_O_2_)(C_18_H_40_N_4_)]ClO_4_
*M* *_r_* = 536.40
Orthorhombic, 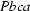

*a* = 17.822 (6) Å
*b* = 12.995 (6) Å
*c* = 22.381 (7) Å
*V* = 5183 (3) Å^3^
*Z* = 8
Mo *K*α radiation
μ = 1.09 mm^−1^
*T* = 153 K
0.30 × 0.11 × 0.04 mm

#### Data collection

Rigaku AFC12/SATURN724 diffractometer
Absorption correction: multi-scan (*ABSCOR*; Higashi, 1995 ▶) *T*_min_ = 0.602, *T*_max_ = 1.000
23785 measured reflections
4534 independent reflections
4080 reflections with *I* > 2σ(*I*)
*R*_int_ = 0.069

#### Refinement

*R*[*F* ^2^ > 2σ(*F* ^2^)] = 0.080
*wR*(*F* ^2^) = 0.183
*S* = 1.29
4534 reflections
320 parameters
22 restraints
H atoms treated by a mixture of independent and constrained refinement
Δρ_max_ = 0.63 e Å^−3^
Δρ_min_ = −0.48 e Å^−3^

### 

Data collection: *CrystalClear* (Molecular Structure Corporation & Rigaku, 2005 ▶); cell refinement: *CrystalClear*; data reduction: *CrystalClear*; program(s) used to solve structure: *SHELXS97* (Sheldrick, 2008 ▶); program(s) used to refine structure: *SHELXL97* (Sheldrick, 2008 ▶); molecular graphics: *ORTEP-3* (Farrugia, 1997 ▶) and *DIAMOND* (Brandenburg, 2006 ▶); software used to prepare material for publication: *publCIF* (Westrip, 2010 ▶).

## Supplementary Material

Crystal structure: contains datablock(s) global, I. DOI: 10.1107/S1600536811045582/hb6470sup1.cif

Structure factors: contains datablock(s) I. DOI: 10.1107/S1600536811045582/hb6470Isup2.hkl

Additional supplementary materials:  crystallographic information; 3D view; checkCIF report

## Figures and Tables

**Table 1:** Selected bond lengths (Å)

Zn—O1	1.973 (4)
Zn—N1	2.124 (4)
Zn—N2	2.216 (4)
Zn—N3	2.095 (4)
Zn—N4	2.153 (4)

**Table 2:** Hydrogen-bond geometry (Å, °)

*D*—H⋯*A*	*D*—H	H⋯*A*	*D*⋯*A*	*D*—H⋯*A*
N1—H1n⋯O4	0.88 (4)	2.14 (5)	3.017 (10)	177 (5)
N2—H2n⋯O2^i^	0.88 (4)	2.60 (4)	3.375 (6)	147 (4)
N3—H3n⋯O5	0.88 (3)	2.42 (3)	3.228 (8)	153 (5)
N4—H4n⋯O2	0.88 (3)	2.25 (3)	2.978 (6)	140 (4)

Symmetry code: (i) 
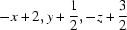
.

**Fig. 1.**
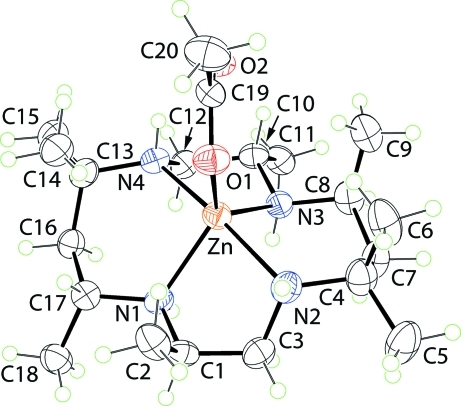
The molecular structure of (I) showing the atom-labelling scheme and displacement ellipsoids at the 50% probability level. The perchlorate counter-ion is omitted.

**Fig. 2.**
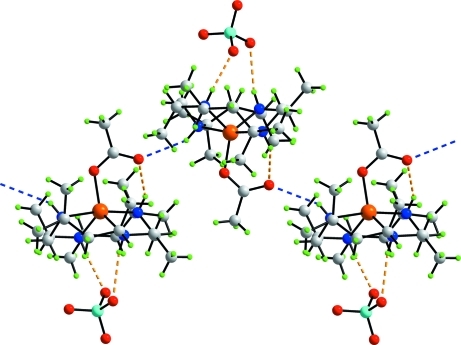
Helical supramolecular chains in (I) sustained by N—H···O(acetate) hydrogen bonds (blue dashed lines). Associated with these chains are the perchlorate anions held in place by N—H···O interactions (orange dashed lines).

## supplementary crystallographic information

### Comment

In continuation of long-terms studies of the synthesis, characterization and biological activities of methyl substituted tetraazamacrocyclic ligands and their metal complexes (Bembi *et al.*, 1990; Roy *et al.*, 2007; Hazari *et al.*, 2008; Roy *et al.*, 2011), the synthesis and crystal structure of the title complex, (I), was investigated.

The asymmetric unit of (I) comprises a ZnL(O_2_CMe) cation, Fig. 1, and a disordered perchlorate anion; *L* = 2,5,5,7,9,12,12,14-octamethyl-1,4,8,11-tetra-azacyclotetradecane. The Zn atom is five-coordinate, Table 1, within an N_4_O donor set derived from the four N atoms of the macrocyclic ligand and an O atom of a monodentate acetate; the Zn···O2 separation is 3.132 (4) Å. Based on the value of τ = 0.71, compared with the values of t = 0.0 and 1.0 for ideal square pyramidal and trigonal bipyramidal, respectively (Addison *et al.*, 1984), the coordination geometry is distorted trigonal bipyramidal with the N2 and N4 atoms occupying axial positions; N2—Zn—N4 = 170.89 (16)°.

The cations in (I) are connected *via* weak *N*—*H*···*O*(acetate) hydrogen bonds, Table 1, which stabilize supramolecular helical chains along the *b* axis, Fig. 2; intramolecular *N*—*H*···*O*(acetate) hydrogen bonds are also present. The perchlorate anions associate with the chains *via* N—H···O interactions, also shown in Fig. 2.

### Experimental

The title complex, (I), was prepared by the anion exchange reaction of [ZnL(O_2_CMe)][O_2_CMe] with perchlorate, where *L* is 2,5,5,7,9,12,12,14-octamethyl-1,4,8,11-tetra-azacyclotetradecane. Thus, [ZnL(O_2_CMe)][O_2_CMe] (0.495 g, 1.0 mmol) was dissolved in hot methanol (40 ml) and sodium perchlorate hexahydrate (0.460 g, 2.0 mmol) added. The reaction mixture was heated for 15 min. During heating a white product separated out. After cooling at room temperature for 30 min, the product, (I), was filtered off, washed with methanol followed by diethyl ether and dried in a desiccator over silica-gel. The yield was about 40%. Anal. Calc for C_20_H_43_ClN_4_O_6_Zn: C, 44.78; H, 7.46; N, 10.45; Zn, 12.20%. Found: C, 44.63; H, 7.32; N, 10.25; Zn, 12.01%. IR (cm^-1^): 1599 ν(O_2_C); 1129, 624 ν(ClO_4_). Colourless prisms of (I) were obtained from slow evaporation of its methanol solution.

### Refinement

The H-atoms were placed in calculated positions (C—H = 0.98–1.00 Å) and were included in the refinement in the riding model approximation, with *U*_iso_(H) set to 1.2–1.5*U*_equiv_(C). The N—H atoms were located from a difference map and refined with N—H = 0.88±0.01 Å, and with *U*_iso_(H) set to 1.2*U*_equiv_(N). The perchlorate anion was disordered about a pseudo-threefold axis. Two positions were resolved for three O atoms. The Cl—O and O···O distances were constrained to 1.44±0.01 and 2.34±0.01 Å, respectively. From anisotropic refinement, the major component of the disordered residue had a site occupancy factor = 0.692 (11).

### Crystal data

**Table:**

[Zn(C_2_H_3_O_2_)(C_18_H_40_N_4_)]ClO_4_	*F*(000) = 2288
*M**_r_* = 536.40	*D*_x_ = 1.375 Mg m^−^^3^
Orthorhombic, *P**b**c**a*	Mo *K*α radiation, λ = 0.71073 Å
Hall symbol: -P 2ac 2ab	Cell parameters from 7295 reflections
*a* = 17.822 (6) Å	θ = 2.9–30.3°
*b* = 12.995 (6) Å	µ = 1.09 mm^−^^1^
*c* = 22.381 (7) Å	*T* = 153 K
*V* = 5183 (3) Å^3^	Prism, colourless
*Z* = 8	0.30 × 0.11 × 0.04 mm

### Data collection

**Table:**

Rigaku AFC12K/SATURN724 diffractometer	4534 independent reflections
Radiation source: fine-focus sealed tube	4080 reflections with *I* > 2σ(*I*)
graphite	*R*_int_ = 0.069
ω scans	θ_max_ = 25.0°, θ_min_ = 2.8°
Absorption correction: multi-scan (*ABSCOR*; Higashi, 1995)	*h* = −20→19
*T*_min_ = 0.602, *T*_max_ = 1.000	*k* = −12→15
23785 measured reflections	*l* = −23→26

### Refinement

**Table:**

Refinement on *F*^2^	Primary atom site location: structure-invariant direct methods
Least-squares matrix: full	Secondary atom site location: difference Fourier map
*R*[*F*^2^ > 2σ(*F*^2^)] = 0.080	Hydrogen site location: inferred from neighbouring sites
*wR*(*F*^2^) = 0.183	H atoms treated by a mixture of independent and constrained refinement
*S* = 1.29	*w* = 1/[σ^2^(*F*_o_^2^) + (0.0546*P*)^2^ + 10.5654*P*] where *P* = (*F*_o_^2^ + 2*F*_c_^2^)/3
4534 reflections	(Δ/σ)_max_ = 0.002
320 parameters	Δρ_max_ = 0.63 e Å^−^^3^
22 restraints	Δρ_min_ = −0.48 e Å^−^^3^

### Special details

**Table:**

Geometry. All s.u.'s (except the s.u. in the dihedral angle between two l.s. planes) are estimated using the full covariance matrix. The cell s.u.'s are taken into account individually in the estimation of s.u.'s in distances, angles and torsion angles; correlations between s.u.'s in cell parameters are only used when they are defined by crystal symmetry. An approximate (isotropic) treatment of cell s.u.'s is used for estimating s.u.'s involving l.s. planes.
Refinement. Refinement of *F*^2^ against ALL reflections. The weighted *R*-factor *wR* and goodness of fit *S* are based on *F*^2^, conventional *R*-factors *R* are based on *F*, with *F* set to zero for negative *F*^2^. The threshold expression of *F*^2^ > 2σ(*F*^2^) is used only for calculating *R*-factors(gt) *etc*. and is not relevant to the choice of reflections for refinement. *R*-factors based on *F*^2^ are statistically about twice as large as those based on *F*, and *R*- factors based on ALL data will be even larger.

### Fractional atomic coordinates and isotropic or equivalent isotropic displacement parameters (Å^2^)

**Table:**

	*x*	*y*	*z*	*U*_iso_*/*U*_eq_	Occ. (<1)
Zn	1.01476 (3)	0.24442 (4)	0.65431 (3)	0.0345 (2)	
O1	1.0503 (2)	0.2678 (3)	0.73667 (16)	0.0433 (9)	
O2	1.0583 (2)	0.1015 (3)	0.76154 (16)	0.0491 (10)	
N1	1.0563 (2)	0.3315 (3)	0.58108 (19)	0.0335 (9)	
H1N	1.032 (3)	0.304 (4)	0.5507 (16)	0.040*	
N2	0.9363 (2)	0.3757 (3)	0.6641 (2)	0.0392 (10)	
H2N	0.952 (3)	0.419 (3)	0.6913 (19)	0.047*	
N3	0.9287 (2)	0.1464 (3)	0.62552 (19)	0.0349 (10)	
H3N	0.923 (3)	0.166 (4)	0.5881 (9)	0.042*	
N4	1.0846 (2)	0.1156 (3)	0.63040 (19)	0.0351 (10)	
H4N	1.093 (3)	0.086 (4)	0.6652 (12)	0.042*	
C1	1.0292 (3)	0.4397 (4)	0.5913 (2)	0.0379 (12)	
H1	1.0340	0.4792	0.5531	0.046*	
C2	1.0757 (3)	0.4927 (4)	0.6390 (3)	0.0444 (14)	
H2A	1.0825	0.4461	0.6730	0.067*	
H2B	1.0498	0.5552	0.6524	0.067*	
H2C	1.1248	0.5111	0.6225	0.067*	
C3	0.9465 (3)	0.4332 (4)	0.6080 (2)	0.0404 (13)	
H3A	0.9259	0.5035	0.6126	0.048*	
H3B	0.9185	0.3985	0.5755	0.048*	
C4	0.8573 (3)	0.3561 (5)	0.6842 (3)	0.0467 (14)	
C5	0.8056 (4)	0.4482 (5)	0.6709 (4)	0.0645 (19)	
H5A	0.8290	0.5115	0.6856	0.097*	
H5B	0.7572	0.4380	0.6909	0.097*	
H5C	0.7976	0.4535	0.6277	0.097*	
C6	0.8606 (4)	0.3452 (5)	0.7522 (3)	0.0587 (17)	
H6A	0.8995	0.2952	0.7629	0.088*	
H6B	0.8119	0.3214	0.7670	0.088*	
H6C	0.8725	0.4121	0.7701	0.088*	
C7	0.8231 (3)	0.2632 (4)	0.6516 (3)	0.0477 (15)	
H7A	0.8237	0.2799	0.6084	0.057*	
H7B	0.7697	0.2597	0.6636	0.057*	
C8	0.8554 (3)	0.1523 (4)	0.6580 (3)	0.0439 (13)	
H8	0.8203	0.1065	0.6354	0.053*	
C9	0.8603 (3)	0.1078 (5)	0.7196 (3)	0.0535 (16)	
H9A	0.8657	0.0329	0.7169	0.080*	
H9B	0.8146	0.1247	0.7418	0.080*	
H9C	0.9039	0.1368	0.7403	0.080*	
C10	0.9591 (3)	0.0393 (4)	0.6200 (2)	0.0369 (12)	
H10	0.9632	0.0086	0.6608	0.044*	
C11	0.9102 (3)	−0.0302 (4)	0.5816 (3)	0.0435 (13)	
H11A	0.8634	−0.0454	0.6028	0.065*	
H11B	0.9370	−0.0945	0.5734	0.065*	
H11C	0.8987	0.0046	0.5438	0.065*	
C12	1.0379 (3)	0.0472 (4)	0.5929 (2)	0.0372 (12)	
H12A	1.0610	−0.0220	0.5907	0.045*	
H12B	1.0347	0.0752	0.5518	0.045*	
C13	1.1625 (3)	0.1373 (4)	0.6071 (2)	0.0397 (12)	
C14	1.2063 (3)	0.1843 (5)	0.6585 (3)	0.0494 (15)	
H14A	1.2567	0.2030	0.6448	0.074*	
H14B	1.2101	0.1343	0.6911	0.074*	
H14C	1.1802	0.2461	0.6728	0.074*	
C15	1.2013 (3)	0.0373 (5)	0.5876 (3)	0.0500 (15)	
H15A	1.2539	0.0515	0.5778	0.075*	
H15B	1.1758	0.0094	0.5524	0.075*	
H15C	1.1989	−0.0129	0.6202	0.075*	
C16	1.1590 (3)	0.2100 (4)	0.5530 (2)	0.0359 (12)	
H16A	1.1229	0.1803	0.5242	0.043*	
H16B	1.2089	0.2089	0.5336	0.043*	
C17	1.1374 (3)	0.3234 (4)	0.5624 (2)	0.0352 (12)	
H17	1.1693	0.3523	0.5951	0.042*	
C18	1.1531 (3)	0.3833 (4)	0.5052 (2)	0.0449 (13)	
H18A	1.2058	0.3746	0.4940	0.067*	
H18B	1.1426	0.4564	0.5118	0.067*	
H18C	1.1209	0.3573	0.4731	0.067*	
C19	1.0657 (3)	0.1956 (4)	0.7740 (2)	0.0390 (12)	
C20	1.0900 (4)	0.2282 (5)	0.8341 (3)	0.0555 (16)	
H20A	1.0463	0.2319	0.8606	0.083*	
H20B	1.1137	0.2961	0.8316	0.083*	
H20C	1.1260	0.1783	0.8500	0.083*	
Cl	0.90070 (8)	0.24995 (10)	0.45914 (6)	0.0454 (4)	0.692 (11)
O3	0.8898 (3)	0.2216 (4)	0.39872 (16)	0.0672 (13)	0.692 (11)
O4	0.9752 (3)	0.2296 (10)	0.4786 (4)	0.102 (4)	0.692 (11)
O5	0.8516 (5)	0.1844 (6)	0.4969 (3)	0.081 (3)	0.692 (11)
O6	0.8776 (6)	0.3529 (4)	0.4698 (3)	0.074 (3)	0.692 (11)
Cl1	0.90070 (8)	0.24995 (10)	0.45914 (6)	0.0454 (4)	0.308 (11)
O13	0.8898 (3)	0.2216 (4)	0.39872 (16)	0.0672 (13)	0.308 (11)
O14	0.9755 (6)	0.2979 (15)	0.4630 (7)	0.102 (4)	0.308 (11)
O15	0.8985 (12)	0.1657 (9)	0.4985 (5)	0.081 (3)	0.308 (11)
O16	0.8483 (9)	0.3283 (11)	0.4765 (7)	0.074 (3)	0.308 (11)

### Atomic displacement parameters (Å^2^)

**Table:**

	*U*^11^	*U*^22^	*U*^33^	*U*^12^	*U*^13^	*U*^23^
Zn	0.0378 (4)	0.0312 (4)	0.0344 (4)	−0.0006 (3)	0.0006 (2)	0.0000 (2)
O1	0.054 (2)	0.042 (2)	0.034 (2)	0.0042 (18)	−0.0023 (17)	−0.0004 (17)
O2	0.071 (3)	0.038 (2)	0.038 (2)	−0.006 (2)	−0.0047 (19)	0.0031 (18)
N1	0.036 (2)	0.029 (2)	0.035 (2)	0.0017 (19)	−0.0041 (18)	−0.0017 (18)
N2	0.039 (3)	0.036 (3)	0.042 (3)	0.003 (2)	0.001 (2)	−0.004 (2)
N3	0.036 (2)	0.032 (2)	0.037 (2)	0.0009 (18)	0.0018 (19)	−0.0004 (19)
N4	0.038 (2)	0.033 (2)	0.034 (2)	0.0042 (19)	−0.0036 (19)	0.0014 (19)
C1	0.053 (3)	0.025 (3)	0.036 (3)	0.004 (2)	0.004 (2)	0.000 (2)
C2	0.053 (4)	0.033 (3)	0.047 (3)	−0.008 (3)	0.012 (3)	−0.009 (2)
C3	0.044 (3)	0.037 (3)	0.041 (3)	0.008 (2)	−0.001 (2)	−0.005 (2)
C4	0.044 (3)	0.041 (3)	0.055 (4)	0.000 (3)	0.007 (3)	−0.008 (3)
C5	0.049 (4)	0.045 (4)	0.100 (5)	0.009 (3)	0.005 (4)	−0.014 (4)
C6	0.058 (4)	0.057 (4)	0.060 (4)	−0.013 (3)	0.024 (3)	−0.017 (3)
C7	0.034 (3)	0.050 (4)	0.059 (4)	0.000 (3)	0.004 (3)	−0.013 (3)
C8	0.044 (3)	0.034 (3)	0.053 (3)	−0.005 (2)	0.006 (3)	−0.009 (3)
C9	0.053 (4)	0.052 (4)	0.056 (4)	−0.008 (3)	0.014 (3)	−0.005 (3)
C10	0.044 (3)	0.028 (3)	0.038 (3)	0.001 (2)	−0.003 (2)	0.001 (2)
C11	0.051 (3)	0.032 (3)	0.047 (3)	−0.006 (3)	0.005 (3)	−0.003 (2)
C12	0.039 (3)	0.034 (3)	0.038 (3)	0.004 (2)	0.000 (2)	−0.005 (2)
C13	0.037 (3)	0.036 (3)	0.046 (3)	0.005 (2)	−0.003 (2)	−0.002 (2)
C14	0.046 (3)	0.054 (4)	0.048 (4)	−0.002 (3)	−0.006 (3)	0.004 (3)
C15	0.044 (3)	0.045 (3)	0.061 (4)	0.008 (3)	0.002 (3)	0.002 (3)
C16	0.034 (3)	0.035 (3)	0.039 (3)	0.001 (2)	0.000 (2)	−0.005 (2)
C17	0.038 (3)	0.035 (3)	0.032 (3)	−0.001 (2)	0.000 (2)	−0.004 (2)
C18	0.049 (3)	0.037 (3)	0.049 (3)	−0.003 (3)	0.005 (3)	0.002 (3)
C19	0.038 (3)	0.044 (3)	0.035 (3)	0.002 (2)	0.007 (2)	0.010 (2)
C20	0.076 (5)	0.046 (4)	0.045 (3)	−0.008 (3)	−0.006 (3)	0.001 (3)
Cl	0.0548 (9)	0.0363 (7)	0.0450 (8)	0.0057 (6)	−0.0117 (6)	−0.0036 (6)
O3	0.090 (4)	0.066 (3)	0.045 (3)	0.003 (3)	−0.022 (2)	−0.010 (2)
O4	0.060 (4)	0.161 (11)	0.085 (6)	0.047 (6)	−0.037 (4)	−0.066 (7)
O5	0.103 (8)	0.070 (5)	0.069 (4)	−0.023 (5)	−0.008 (5)	0.019 (3)
O6	0.122 (8)	0.025 (4)	0.076 (4)	0.008 (4)	−0.001 (4)	0.001 (3)
Cl1	0.0548 (9)	0.0363 (7)	0.0450 (8)	0.0057 (6)	−0.0117 (6)	−0.0036 (6)
O13	0.090 (4)	0.066 (3)	0.045 (3)	0.003 (3)	−0.022 (2)	−0.010 (2)
O14	0.060 (4)	0.161 (11)	0.085 (6)	0.047 (6)	−0.037 (4)	−0.066 (7)
O15	0.103 (8)	0.070 (5)	0.069 (4)	−0.023 (5)	−0.008 (5)	0.019 (3)
O16	0.122 (8)	0.025 (4)	0.076 (4)	0.008 (4)	−0.001 (4)	0.001 (3)

### Geometric parameters (Å, °)

**Table:**

Zn—O1	1.973 (4)	C9—H9A	0.9800
Zn—N1	2.124 (4)	C9—H9B	0.9800
Zn—N2	2.216 (4)	C9—H9C	0.9800
Zn—N3	2.095 (4)	C10—C11	1.521 (7)
Zn—N4	2.153 (4)	C10—C12	1.534 (7)
O1—C19	1.286 (6)	C10—H10	1.0000
O2—C19	1.261 (7)	C11—H11A	0.9800
N1—C1	1.504 (6)	C11—H11B	0.9800
N1—C17	1.508 (6)	C11—H11C	0.9800
N1—H1N	0.882 (10)	C12—H12A	0.9900
N2—C3	1.473 (7)	C12—H12B	0.9900
N2—C4	1.500 (7)	C13—C14	1.518 (8)
N2—H2N	0.878 (10)	C13—C16	1.537 (7)
N3—C8	1.497 (7)	C13—C15	1.535 (8)
N3—C10	1.499 (6)	C14—H14A	0.9800
N3—H3N	0.879 (10)	C14—H14B	0.9800
N4—C12	1.478 (7)	C14—H14C	0.9800
N4—C13	1.511 (7)	C15—H15A	0.9800
N4—H4N	0.880 (10)	C15—H15B	0.9800
C1—C2	1.518 (7)	C15—H15C	0.9800
C1—C3	1.523 (8)	C16—C17	1.537 (7)
C1—H1	1.0000	C16—H16A	0.9900
C2—H2A	0.9800	C16—H16B	0.9900
C2—H2B	0.9800	C17—C18	1.524 (7)
C2—H2C	0.9800	C17—H17	1.0000
C3—H3A	0.9900	C18—H18A	0.9800
C3—H3B	0.9900	C18—H18B	0.9800
C4—C6	1.528 (9)	C18—H18C	0.9800
C4—C7	1.537 (8)	C19—C20	1.476 (8)
C4—C5	1.540 (8)	C20—H20A	0.9800
C5—H5A	0.9800	C20—H20B	0.9800
C5—H5B	0.9800	C20—H20C	0.9800
C5—H5C	0.9800	Cl—O6	1.420 (5)
C6—H6A	0.9800	Cl—O3	1.415 (4)
C6—H6B	0.9800	Cl—O4	1.422 (5)
C6—H6C	0.9800	Cl—O5	1.484 (5)
C7—C8	1.559 (8)	Cl1—O15	1.405 (7)
C7—H7A	0.9900	Cl1—O16	1.435 (7)
C7—H7B	0.9900	Cl1—O13	1.415 (4)
C8—C9	1.497 (8)	Cl1—O14	1.474 (8)
C8—H8	1.0000		
			
O1—Zn—N3	128.04 (17)	C7—C8—H8	105.8
O1—Zn—N1	121.79 (16)	C8—C9—H9A	109.5
N3—Zn—N1	109.96 (17)	C8—C9—H9B	109.5
O1—Zn—N4	99.58 (16)	H9A—C9—H9B	109.5
N3—Zn—N4	82.75 (16)	C8—C9—H9C	109.5
N1—Zn—N4	91.23 (16)	H9A—C9—H9C	109.5
O1—Zn—N2	89.52 (16)	H9B—C9—H9C	109.5
N3—Zn—N2	92.08 (17)	N3—C10—C11	113.0 (4)
N1—Zn—N2	83.46 (17)	N3—C10—C12	107.5 (4)
N4—Zn—N2	170.89 (16)	C11—C10—C12	109.9 (4)
C19—O1—Zn	124.3 (4)	N3—C10—H10	108.7
C1—N1—C17	114.5 (4)	C11—C10—H10	108.7
C1—N1—Zn	105.6 (3)	C12—C10—H10	108.7
C17—N1—Zn	120.6 (3)	C10—C11—H11A	109.5
C1—N1—H1N	109 (4)	C10—C11—H11B	109.5
C17—N1—H1N	104 (4)	H11A—C11—H11B	109.5
Zn—N1—H1N	102 (4)	C10—C11—H11C	109.5
C3—N2—C4	117.2 (4)	H11A—C11—H11C	109.5
C3—N2—Zn	103.2 (3)	H11B—C11—H11C	109.5
C4—N2—Zn	119.4 (3)	N4—C12—C10	109.3 (4)
C3—N2—H2N	103 (4)	N4—C12—H12A	109.8
C4—N2—H2N	102 (4)	C10—C12—H12A	109.8
Zn—N2—H2N	111 (4)	N4—C12—H12B	109.8
C8—N3—C10	113.7 (4)	C10—C12—H12B	109.8
C8—N3—Zn	117.3 (3)	H12A—C12—H12B	108.3
C10—N3—Zn	109.0 (3)	N4—C13—C14	106.6 (4)
C8—N3—H3N	110 (4)	N4—C13—C16	110.4 (4)
C10—N3—H3N	103 (4)	C14—C13—C16	111.8 (5)
Zn—N3—H3N	102 (4)	N4—C13—C15	110.7 (4)
C12—N4—C13	115.7 (4)	C14—C13—C15	109.0 (5)
C12—N4—Zn	106.5 (3)	C16—C13—C15	108.4 (4)
C13—N4—Zn	118.1 (3)	C13—C14—H14A	109.5
C12—N4—H4N	109 (4)	C13—C14—H14B	109.5
C13—N4—H4N	104 (4)	H14A—C14—H14B	109.5
Zn—N4—H4N	102 (4)	C13—C14—H14C	109.5
N1—C1—C2	110.9 (4)	H14A—C14—H14C	109.5
N1—C1—C3	107.2 (4)	H14B—C14—H14C	109.5
C2—C1—C3	112.4 (4)	C13—C15—H15A	109.5
N1—C1—H1	108.8	C13—C15—H15B	109.5
C2—C1—H1	108.8	H15A—C15—H15B	109.5
C3—C1—H1	108.8	C13—C15—H15C	109.5
C1—C2—H2A	109.5	H15A—C15—H15C	109.5
C1—C2—H2B	109.5	H15B—C15—H15C	109.5
H2A—C2—H2B	109.5	C13—C16—C17	119.5 (4)
C1—C2—H2C	109.5	C13—C16—H16A	107.5
H2A—C2—H2C	109.5	C17—C16—H16A	107.5
H2B—C2—H2C	109.5	C13—C16—H16B	107.5
N2—C3—C1	110.9 (4)	C17—C16—H16B	107.5
N2—C3—H3A	109.5	H16A—C16—H16B	107.0
C1—C3—H3A	109.5	N1—C17—C18	111.9 (4)
N2—C3—H3B	109.5	N1—C17—C16	110.2 (4)
C1—C3—H3B	109.5	C18—C17—C16	109.2 (4)
H3A—C3—H3B	108.1	N1—C17—H17	108.5
N2—C4—C6	106.2 (5)	C18—C17—H17	108.5
N2—C4—C7	111.3 (4)	C16—C17—H17	108.5
C6—C4—C7	114.6 (5)	C17—C18—H18A	109.5
N2—C4—C5	111.8 (5)	C17—C18—H18B	109.5
C6—C4—C5	106.7 (5)	H18A—C18—H18B	109.5
C7—C4—C5	106.3 (5)	C17—C18—H18C	109.5
C4—C5—H5A	109.5	H18A—C18—H18C	109.5
C4—C5—H5B	109.5	H18B—C18—H18C	109.5
H5A—C5—H5B	109.5	O2—C19—O1	122.8 (5)
C4—C5—H5C	109.5	O2—C19—C20	120.7 (5)
H5A—C5—H5C	109.5	O1—C19—C20	116.4 (5)
H5B—C5—H5C	109.5	C19—C20—H20A	109.5
C4—C6—H6A	109.5	C19—C20—H20B	109.5
C4—C6—H6B	109.5	H20A—C20—H20B	109.5
H6A—C6—H6B	109.5	C19—C20—H20C	109.5
C4—C6—H6C	109.5	H20A—C20—H20C	109.5
H6A—C6—H6C	109.5	H20B—C20—H20C	109.5
H6B—C6—H6C	109.5	O6—Cl—O3	111.5 (4)
C4—C7—C8	122.4 (5)	O6—Cl—O4	113.3 (4)
C4—C7—H7A	106.7	O3—Cl—O4	111.8 (4)
C8—C7—H7A	106.7	O6—Cl—O5	106.0 (4)
C4—C7—H7B	106.7	O3—Cl—O5	108.3 (4)
C8—C7—H7B	106.7	O4—Cl—O5	105.6 (4)
H7A—C7—H7B	106.6	O15—Cl1—O16	111.4 (7)
N3—C8—C9	112.1 (5)	O15—Cl1—O13	113.1 (6)
N3—C8—C7	108.9 (4)	O16—Cl1—O13	110.8 (6)
C9—C8—C7	117.6 (5)	O15—Cl1—O14	108.5 (6)
N3—C8—H8	105.8	O16—Cl1—O14	105.8 (6)
C9—C8—H8	105.8	O13—Cl1—O14	106.9 (6)
			
N3—Zn—O1—C19	48.1 (5)	C3—N2—C4—C6	−153.3 (5)
N1—Zn—O1—C19	−137.6 (4)	Zn—N2—C4—C6	80.8 (5)
N4—Zn—O1—C19	−40.2 (4)	C3—N2—C4—C7	81.4 (6)
N2—Zn—O1—C19	140.4 (4)	Zn—N2—C4—C7	−44.5 (6)
O1—Zn—N1—C1	−66.6 (4)	C3—N2—C4—C5	−37.3 (7)
N3—Zn—N1—C1	108.6 (3)	Zn—N2—C4—C5	−163.1 (4)
N4—Zn—N1—C1	−168.7 (3)	N2—C4—C7—C8	63.1 (7)
N2—Zn—N1—C1	18.7 (3)	C6—C4—C7—C8	−57.4 (7)
O1—Zn—N1—C17	65.2 (4)	C5—C4—C7—C8	−175.0 (5)
N3—Zn—N1—C17	−119.6 (4)	C10—N3—C8—C9	57.6 (6)
N4—Zn—N1—C17	−36.9 (4)	Zn—N3—C8—C9	−71.3 (5)
N2—Zn—N1—C17	150.5 (4)	C10—N3—C8—C7	−170.5 (4)
O1—Zn—N2—C3	134.4 (3)	Zn—N3—C8—C7	60.6 (5)
N3—Zn—N2—C3	−97.6 (3)	C4—C7—C8—N3	−71.8 (7)
N1—Zn—N2—C3	12.3 (3)	C4—C7—C8—C9	57.1 (8)
O1—Zn—N2—C4	−93.4 (4)	C8—N3—C10—C11	65.2 (6)
N3—Zn—N2—C4	34.7 (4)	Zn—N3—C10—C11	−161.8 (4)
N1—Zn—N2—C4	144.5 (4)	C8—N3—C10—C12	−173.3 (4)
O1—Zn—N3—C8	48.5 (4)	Zn—N3—C10—C12	−40.3 (5)
N1—Zn—N3—C8	−126.3 (4)	C13—N4—C12—C10	−175.8 (4)
N4—Zn—N3—C8	145.0 (4)	Zn—N4—C12—C10	−42.4 (5)
N2—Zn—N3—C8	−42.5 (4)	N3—C10—C12—N4	56.2 (5)
O1—Zn—N3—C10	−82.6 (4)	C11—C10—C12—N4	179.6 (4)
N1—Zn—N3—C10	102.6 (3)	C12—N4—C13—C14	−165.8 (4)
N4—Zn—N3—C10	13.9 (3)	Zn—N4—C13—C14	66.3 (5)
N2—Zn—N3—C10	−173.6 (3)	C12—N4—C13—C16	72.6 (5)
O1—Zn—N4—C12	143.2 (3)	Zn—N4—C13—C16	−55.3 (5)
N3—Zn—N4—C12	15.7 (3)	C12—N4—C13—C15	−47.5 (6)
N1—Zn—N4—C12	−94.3 (3)	Zn—N4—C13—C15	−175.3 (3)
O1—Zn—N4—C13	−84.7 (4)	N4—C13—C16—C17	70.6 (6)
N3—Zn—N4—C13	147.8 (4)	C14—C13—C16—C17	−47.8 (6)
N1—Zn—N4—C13	37.8 (4)	C15—C13—C16—C17	−167.9 (5)
C17—N1—C1—C2	−58.5 (5)	C1—N1—C17—C18	−58.2 (6)
Zn—N1—C1—C2	76.7 (4)	Zn—N1—C17—C18	174.0 (3)
C17—N1—C1—C3	178.5 (4)	C1—N1—C17—C16	−179.8 (4)
Zn—N1—C1—C3	−46.3 (4)	Zn—N1—C17—C16	52.3 (5)
C4—N2—C3—C1	−175.7 (4)	C13—C16—C17—N1	−68.1 (6)
Zn—N2—C3—C1	−42.2 (5)	C13—C16—C17—C18	168.7 (5)
N1—C1—C3—N2	62.6 (5)	Zn—O1—C19—O2	−1.1 (7)
C2—C1—C3—N2	−59.5 (6)	Zn—O1—C19—C20	−178.7 (4)

### Hydrogen-bond geometry (Å, °)

**Table:**

*D*—H···*A*	*D*—H	H···*A*	*D*···*A*	*D*—H···*A*
N1—H1n···O4	0.88 (4)	2.14 (5)	3.017 (10)	177 (5)
N2—H2n···O2^i^	0.88 (4)	2.60 (4)	3.375 (6)	147 (4)
N3—H3n···O5	0.88 (3)	2.42 (3)	3.228 (8)	153 (5)
N4—H4n···O2	0.88 (3)	2.25 (3)	2.978 (6)	140 (4)

Symmetry codes: (i) −*x*+2, *y*+1/2, −*z*+3/2.

## References

[bb1] Addison, A. W., Rao, T. N., Reedijk, J., van Rijn, J. & Verschoor, G. C. (1984). *J. Chem. Soc. Dalton Trans.* pp. 1349–1356.

[bb2] Bembi, R., Roy, T. G., Jhanji, A. K. & Maheswari, A. (1990). *J. Chem. Soc. Dalton Trans.* pp. 3531–3534.

[bb3] Brandenburg, K. (2006). *DIAMOND* Crystal Impact GbR, Bonn, Germany.

[bb4] Farrugia, L. J. (1997). *J. Appl. Cryst.* **30**, 565.

[bb5] Hazari, S. K. S., Roy, S. K. S., Barua, K. K. & Tiekink, E. R. T. (2008). *J. Chem. Crystallogr.* **38**, 1–8.

[bb6] Higashi, T. (1995). *ABSCOR* Rigaku Corporation, Tokyo, Japan.

[bb7] Molecular Structure Corporation & Rigaku (2005). *CrystalClear* MSC, The Woodlands, Texas, USA, and Rigaku Corporation, Tokyo, Japan.

[bb8] Roy, T. G., Hazari, S. K. S., Dey, B. K., Miah, H. A., Olbrich, F. & Rehder, D. (2007). *Inorg. Chem.* **46**, 5372–5380. 10.1021/ic061700t 17539627

[bb9] Roy, T. G., Hazari, S. K. S., Dey, B. K., Nath, B. C., Dutta, A., Olbrich, F. & Rehder, D. (2011). *Inorg. Chim. Acta*, **371**, 63–70.

[bb10] Sheldrick, G. M. (2008). *Acta Cryst.* A**64**, 112–122. 10.1107/S0108767307043930 18156677

[bb11] Westrip, S. P. (2010). *J. Appl. Cryst.* **43**, 920–925.

